# Marine plant mediated green synthesis of silver nanoparticles using mangrove *Rhizophora* *stylosa*: Effect of variable process and their antibacterial activity

**DOI:** 10.12688/f1000research.54661.1

**Published:** 2021-08-06

**Authors:** Nancy Willian, Syukri Syukri, Zulhadjri Zulhadjri, Syukri Arief

**Affiliations:** 1 Department of Chemistry, Faculty of Mathematics and Natural Sciences, Andalas University, Kampus Limau Manis, Padang, West Sumatra, 25163, Indonesia; 2 Department of Teaching and Education, Raja Ali Haji Maritime University, Tanjungpinang, Riau Archipelago, 29115, Indonesia

**Keywords:** green synthesis, Rhizophora stylosa, silver nanoparticles, antibacterial activity

## Abstract

**Background: **Most natural plants used in the synthesis of silver nanoparticles are limited to marine plants. To carry out applications, colloidal silver nanoparticles (AgNps) should have appropriate properties such as homogeneous shapes, small and narrow particle size distribution, and long time stability. This study aims to determine the effects of a variable process of AgNps mediated mangrove *Rhizophora stylosa* (RS) leaf extract, and antibacterial activity.

**Methods**: Synthesis of AgNps was carried out by stirring silver nitrate solution with aqueous extract. The characterization of AgNps was carried out using UV-Vis spectrophotometry, X-ray diffraction (XRD), Dynamic Light Scattering (DLS) zetasizer and Transmission Electron Microscopy (TEM). Evaluation of antibacterial activity was carried out on *E* *scherichia coli* and *Staphylococcus aureus.* Reaction conditions such as the concentration of metal ions (0.001 M, 0.005 M, and 0.01 M), extracts (1%, 3%, and 5% v/v), and the reaction time on the size and stability of nanoparticles were also explored.

**Results**: The UV-Vis spectroscopy showed an absorption of colloidal AgNps in a wavelength range of 403–443 nm.  TEM analysis showed that as-synthesized AgNps were spherical in shape with a size range of 5–87 nm. The use of 0.001 M and 0.005 M of Ag ^+^ resulted in a smaller diameter than the synthesized AgNps, using 0.01 M Ag ^+^, in the same extract concentration. The range of zeta potential was -24.9 mV to -27.7 mV. The as-synthesized AgNps were stable for more than one month. The XRD analysis showed four peaks, which were attributed to the face centered cubic crystal structure of metallic silver. The results of the silver nanoparticles synthesis showed good activity on *E* *scherichia coli* and *Staphylococcus aureus*, with an inhibition zone between 4.1–7.2 mm.

**Conclusions:** The AgNps synthesized with RS leaf extract, which is a reducing agent, showed good potential as an antibacterial component.

## Introduction

Nanotechnology has become a new breakthrough in research, as it finds wide application, especially in biomedical fields. ^1^ One of the most studied materials is silver nanoparticles, which have various methods of synthesis from physics, chemistry, and biology techniques. However, most processes have many disadvantages, especially chemical methods, which require the use of hazardous reagents that are not friendly to the environment. Furthermore, an alternative arises in the synthesis of nanoparticles with a biological approach by utilizing phytochemical compounds contained in plants. ^2^ In general, these phytochemical compounds contain hydroxyl groups, which play a crucial role in the formation of silver nanoparticles through the reduction of Ag ^+^ to Ag ^0^.

The green synthesis of nanoparticles relies on the stability of silver nanoparticles of substances that are non-toxic, as well as solvents and reducing agents. The effectiveness of this product is based on the size and shape of the particles. ^3^ To carry out applications, colloidal silver nanoparticles (AgNps) should have appropriate physical and chemical properties such as homogeneous shapes, small and narrow particle size distribution, and long-time stability. The control of nanoparticle growth from agglomeration is very important in the selection of stabilizers, donor metals, polymers, and surfactants since its potential application strongly depends on the stability. In addition, to reduce the hazardous effect on the environment, the use of natural reagents is being considered. The hydroxyl group in the natural polyphenol component acts as a reducing, as well as capping agent. ^4^ By the double-function utilization of natural plants, the reagent used in the reaction could be diminished.

Currently, most of the natural plants used in the synthesis of silver nanoparticles are limited to terrestrial plants. Only a few studies report the use of marine plants. Indonesia as an archipelago has a wealth of marine resources such as mangroves. Its extracts are known to have antimicrobial abilities that have long been used by the community. ^5^ One of them is *Rhizophora stylosa* (RS), which is a member of the *Rhizophoraceae* family in the Southeast Asian mangrove ecosystem. It is known to have several compounds that play an important role in biomedical applications. Some studies report that *Rhizophora* has several biological activities such as antibacterial, antioxidant and anti-cancer. ^6– 9^ Teraxerol, teraxerone (triterpenoids), sitosterol (steroids), protocatechuic acid and isovanillic acid (phenolic compounds), routine, astilbin, catechin (flavonoids) are the main compounds in RS mangrove plants. Those compounds show extraordinary pharmacological activities and are linked with the presence of –OH. ^10, 11^ In this study, RS leaf extract was used as a reducing agent in the production of RS-AgNps as well as a stabilizer.

To the best of our knowledge, this is the first research on RS mediated synthesis of silver nanoparticles. This study aims to investigate the effects of reaction conditions, such as reaction time, the amount of silver solution, and amount of extract, on the properties of fabricated silver nanoparticles. The antibacterial activity of as-synthesized RS AgNps was evaluated against *Escherichia coli* and *Staphylococcus aureus*, which are the main microbes present in wounds. ^12^

## Methods

### Preparation of *Rhizophora stylosa* leaf extract

Fresh leaves of RS were collected from mangrove forest on Bintan Island, Riau Archipelago Indonesia. They were washed with double distilled water (DDW) to remove dust and other impurities. Clean leaves were shade-dried for 10–15 days and then ground to obtain leaf powder. The extraction of RS leaves was done by adding 20 grams of leaf powder into 100 ml of DDW (1:5) followed by heating at 65 ^o^C for 30 minutes with a hotplate stirrer. This mixture was then filtered using Whatman filter paper no. 1. The obtained extract was stored at 4°C for further experiments.

### Synthesis of colloidal silver nanoparticle

Synthesis of silver nanoparticles was carried out by stirring silver nitrate solution with RS leaf extract in a total volume of 50 mL at a constant speed of 500 rpm. The concentration of AgNO _3_ was varied to be 0.001 M, 0.005 M, and 0.01 M, while RS leaf extract was 1%, 3%, and 5% (v/v). The absorbance of the sample was periodically monitored after one, two, four, and six hours of stirring using UV-Vis spectroscopy. Therefore, all samples were stored in a sealed bottle to measure the absorbance after 24, 72, 168, and 720 hours. In this study, some synthesis variables, such as reaction time, extract, and silver precursor concentration were applied to determine the optimum conditions for fabricating stable colloidal silver nanoparticles.

### The characterization

The diameter and zeta potential of RS-AgNps were characterized using Dynamic Light Scattering (DLS) Zetasizer HORIBA (SZ-100) at 25 °C. Absorbance intensity and surface plasmon resonance (SPR) uptake were analyzed using UV-Vis spectroscopy (Shimadzu UV-1800 spectrophotometer) in wavelength range of 200-800 nm at 5 nm intervals. The device was fully controlled by the UV Probe version 2.42 software monitor package. The shape and particle size distribution of silver nanoparticles were calculated on the basis of TEM images (JEOL JEM 1400) processed using J software. X-ray diffraction (Shimadzu XRD-7000s, λ 1.5406 Å operated at a voltage of 30kV and current of 30mA) were used to investigate crystal structures of the samples.

### Antibacterial activity

Antibacterial activity of the as-synthesized RS-AgNps was tested against *Escherichia coli* (Gram negative bacteria) and *Staphylococcus aureus* (Gram positive bacteria) using the agar diffusion method. Firstly, the bacteria were planted in nutrient agar (NA) and then cultivated for 24 hours at 37 °C. The suspended bacteria were transferred into 100 mL NA media and then poured into a petri dish. Sterile cotton with a colloidal nanoparticle sample with a concentration of 100 mg/mL were deposited in the well. DDW and amoxicillin were adopted as negative and positive controls, respectively, and the area of inhibition was measured after 24 hours of precipitation and incubation, tests were carried out in duplicate and the results displayed were averaged.

## Results and discussion

### UV-Vis spectroscopy analysis

The formation of silver nanoparticles in the samples were specifically recognized by color changes from colorless to light yellow right after mixing the silver precursor and leaf extract ( Table 1). It was confirmed by UV-Vis spectrophotometry analysis where AgNps provide specific peaks at a wavelength of about 403–443 nm. This analysis was based on the SPR phenomenon of spherical metallic nanoparticles, which is strongly influenced by shape and size. ^13^

**Table 1.** Wavelength of *Rhizophora stylosa* silver nanoparticles (RS-AgNps).

Wavelength (nm) RS-AgNps									
Reaction Time (h)	0.001 M (1%)	0.001 M (3%)	0.001 M (5%)	0.005 M (1%)	0.005 M (3%)	0.005 M (5%)	0.01 M (1%)	0.01 M (3%)	0.01 M (5%)
0	0	0	0	0	0	0	426	0	0
1	0	0	0	430.5	0	387	422	0	0
2	0	0	0	424.5	401.5	385	433	419	405
4	403.5	0	0	439	408.5	384	437.5	433	422.5
6	416.5	401	364	412	434.5	385	445.5	438	426
24	409	404.5	398	437.5	414.5	384	450	448	437.5
72	406	416.5	416	441	417.5	397.5	446	445	423
168	416	419.5	414	439	418.5	402.5	446	443	443
720	421	419	419	421	419	419	443	446	446

**Figure 1.**
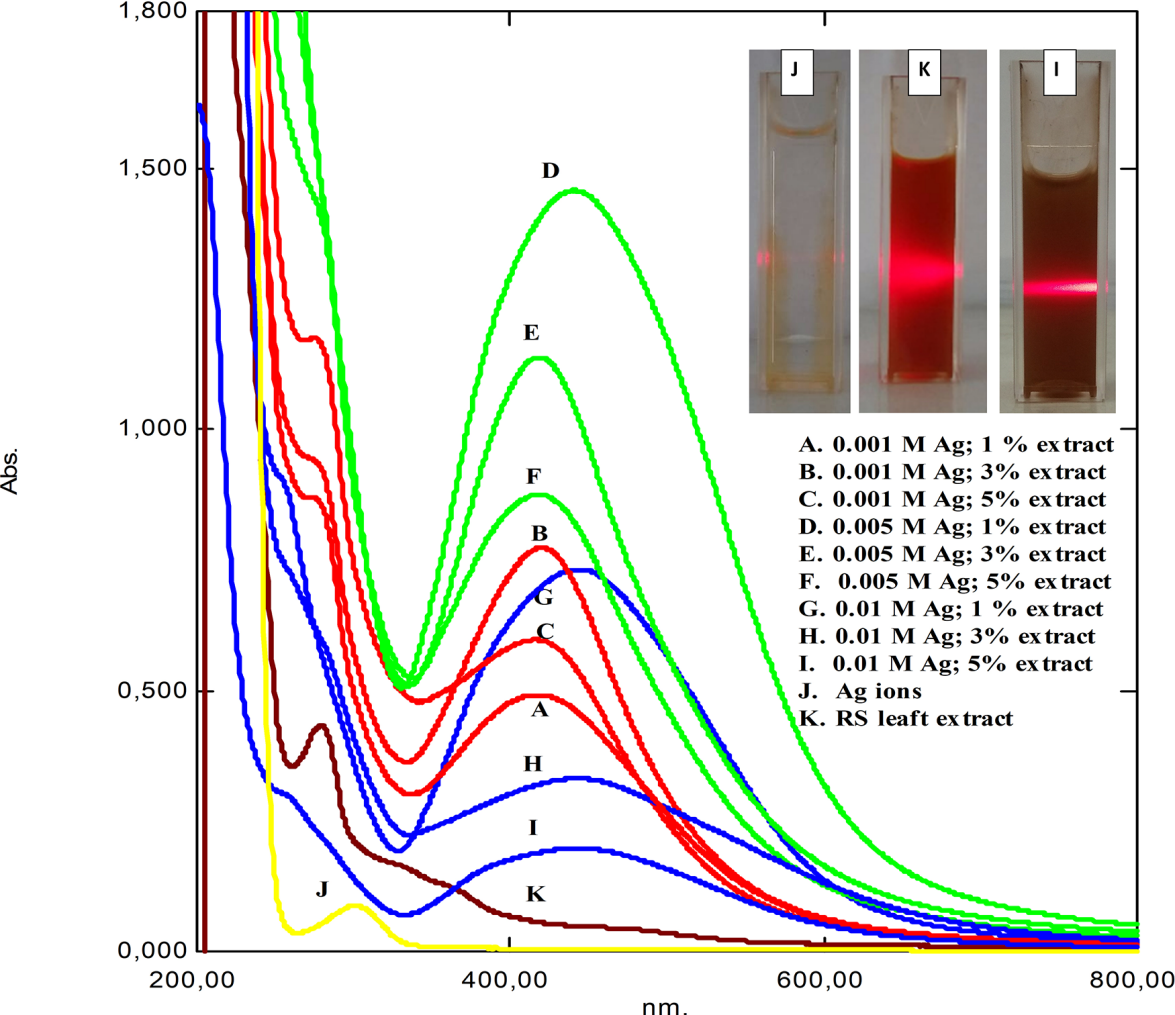
UV-Vis absorption of synthesized *Rhizophora stylosa* silver nanoparticles (RS-AgNps), stabilization Nps after one month. The inset shows the laser beam radiation.

Figure 1 shows the UV-Vis spectrum of as-synthesized RS-AgNps in a varied concentration of AgNO _3_ and RS leaf extract after a one-month reaction. The spectrum of silver nitrate solution (j) and RS leaf extract (k) provided peaks at wavelength 301 and 279 nm, respectively. In general, the peaks were then converted to a wavelength range of 419 to 446 nm, which were specific to spherical AgNps. The inset pictures show laser beam irradiation of the brown-colored samples based on the Tyndal effect. Those results strongly confirmed the formation of metallic silver nanoparticles through the reduction process of Ag ^+^ to Ag ^0^, which consisted of three stages of process i.e. reduction, nucleation, and growth. ^14^ Similar studies on mangrove *Rhizophora lamarckii* plants have shown that spherical AgNps contribute to absorption bands of around 420 nm in the spectrum of particles. ^15^

In this study, RS-AgNps samples were prepared using a varied concentration of silver nitrate (0.001 M, 0.005 M, and 0.01 M) and RS leaf extract (1%, 3%, and 5% v/v) in order to determine the optimum reaction composition. The wavelength and absorbance value based on spectrophotometry analysis are shown in Table 1. It was shown that an increase in extract concentration (1% to 5%) significantly shortened the wavelength, which indicates smaller-sized AgNps were formed. However, based on the wavelength values, AgNps with the addition of 3% leaf extract tended to have less wavelength shift than those of AgNps with 1% and 5%. This suggested that the stability and growth of AgNps were maintained by the addition of 3% extract. This result strongly confirmed that RS leaf extract acted as a capping agent to stabilize and control the growth of AgNps. This was observed in all samples prepared using three different concentrations of silver nitrate.

In addition, an increase in silver nitrate concentration led to longer wavelength and showed the formation of bigger-sized AgNps. This phenomenon was related to the formation of aggregates due to the excessive amount of silver nitrate in the reaction, resulting in bigger sized AgNps. ^16^ Hence, in order to fabricate stable small-sized AgNps, it is sufficient to apply a low concentration of silver nitrate i.e. 0.001 M and 0.005 M.

The leaf extract and silver ion concentration significantly affected the absorption intensity as well, as seen in Table 2. The AgNps samples prepared using 5% extract showed a higher absorption intensity than those prepared using 1% and 3%. This was observed both in the use of 0.001 M and 0.005 M silver nitrate. The absorption intensity reflects the number of metallic nanoparticles formed in the reaction. Hence, these results suggest that the more RS leaf extract used in the reaction, the greater the number of metallic nanoparticles formed. This strongly confirmed the role of leaf extract in reducing Ag ^+^ to Ag ^0^ and the formation of AgNps. This result is beneficial in industry because synthesis is carried out on a large scale.

**Table 2.** Absorbance of *Rhizophora stylosa* silver nanoparticles (RS-AgNps).

	Absorbance (a.u)								
Reaction Time (h)	0.001 M (1%)	0.001 M (3%)	0.001 M (5%)	0.005 M (1%)	0.005 M (3%)	0.005 M (5%)	0.01 M (1%)	0.01 M (3%)	0.01 M (5%)
0	0	0	0	0	0	0	0.433	0	0
1	0	0	0	0.423	0	0.135	0.516	0	0
2	0	0	0	0.574	0.651	0.323	0.567	1.08	1.438
4	0.284	0	0	0.681	0.722	0.661	0.819	1.306	1.727
6	0.359	0.401	0.509	0.927	0.936	0.754	0.941	1.467	1.911
24	0.347	0.413	0.532	0.9669	0.923	0.846	0.971	1.535	2.007
72	0.376	0.491	0.589	1.129	0.967	0.911	0.957	0.765	1.005
168	0.36	0.483	0.587	1.408	0.972	0.876	0.623	0.532	0.943
720	0.352	0.473	0.597	1.458	0.962	0.776	0.723	0.432	0.543

In addition, the samples prepared using 0.005 M silver nitrate solution exhibited higher absorption intensity than those prepared using 0.001 M. It is assumed that the higher concentration of silver nitrate solution provided more reduction and led to a higher number of formed AgNps. However, the excessive silver nitrate solution in a concentration of 0.01 M resulted in a lower absorption intensity.

In general, there was no significant decrease in absorbance intensity during the reaction process up to a one-month reaction. This result suggested that the as-synthesized RS showed good stability. Furthermore, the AgNps size data and its correlation with the concentration of extract and silver solution is supported by the TEM data.

### Transmission electron microscopy, dynamic light scattering, zeta potential and polydispersion index analysis

**Figure 2.**
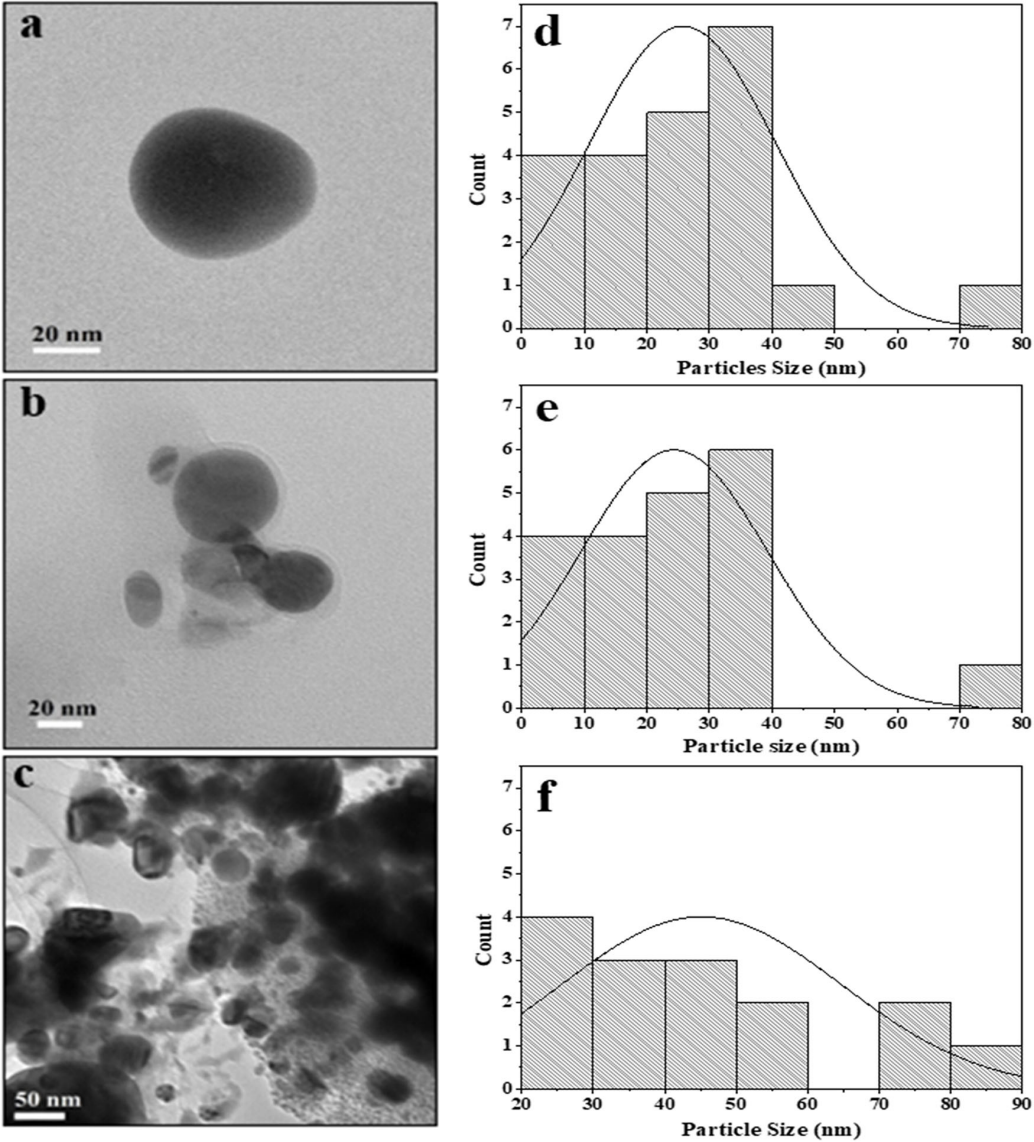
Transmission electron microscope images of *Rhizophora stylosa* silver nanoparticles (RS-AgNps) and particle size distribution: (a, d) AgNps 0.001M 3% with particle size of 5 nm–70 nm and mean diameter 25 nm, (b, e) AgNps 0.005 M 3%, with particle size of 5 nm–77 nm and mean diameter 22 nm (c, f) AgNps 0.01 M 3%, with particle size of 23 nm–87 nm and mean diameter 45 nm.

Figure 2 shows the TEM images of RS-AgNps, which are spherical in shape with a size range of 5–87 nm. The mean diameter of AgNps prepared using 0.001 M, 0.005 M, and 0.01 M silver nitrate and the addition of 3% extract were 25 nm, 22 nm, and 45 nm, respectively. These results show that the concentration of silver nitrate affects the size of the nanoparticles, where the use of 0.01 M silver nitrate resulted in a larger diameter of AgNps than those prepared using a lower concentration. It was confirmed that there is a strong correlation between the mean particle diameter with absorption wavelength of AgNps. The shift to longer wavelength (red shift) in UV-Vis spectrophotometry analysis indicates a larger mean diameter. ^17^ Figure 2a and 2b show smaller particle size.

In Figure 2c, there was a slight agglomeration at 0.01 M. Particles in this study are ionic silver nanoparticles dispersed in aquades, permanent dipole and induced dipole between particles that are close together so that they undergo aggregation due to the van der Waals force. ^18, 19^ Particle aggregation causes the size to become large. Aggregation also indicates the small repulsion force between particles, which causes colloid instability. In previous studies, leaf synthesis for nanoparticles (NP) production in mangrove *Rhizophora mucranata*, ^20^ *Rhizophora apiculata* ^21^ and *Rhizophora lamarckii* ^17^ had successive particle sizes of 60–95, 19–42, 12–28 nm through reduction of silver nitrate compounds. In this study the nanoparticles produced were much smaller in size by controlling the amount of extracts and precursors.

Based on the DLS analysis, the hydrodynamic particle size of colloidal RS-AgNps ranges from 98–125 nm, which is larger than TEM measurements. This is due to the different measurement principles between TEM, and DLS. The latter method measures the hydrodynamic size of nanoparticles in aqueous suspensions including the metal core, and any biological molecule attached to the surface of the particles. ^22^ The zeta potential is an important parameter for assessing the stability of AgNps in aqueous suspensions. When the value is greater, either positive or negative, it suggests that molecules tend to repulse each other, hence it increases the suspension stability. The negative potential values shown by the bio-synthesized AgNps reflect the presence of bio-organic components in the extract as a capping agent. ^13, 23^

A zeta potential value of ± 30 mV is required for indication of a stable nanoparticle suspension. ^24, 25^ The zeta ability of RS-AgNps colloids range from (−24.9) mV up to (−27.7) mV. In addition, the extract concentration in colloidal AgNps influenced the polydispersity index as well. The polydispersity index showed the dimensions of the particle size distribution, while a polydispersity index value > 0.7 indicates a very wide dispersion. ^26^ AgNps 0.001 M and 0.005 M with addition of 3% extract showed a narrow size distribution ( Figure 2d and 2e). It was confirmed by a polydispersity index of 0.37. Similar research was carried out by Nasiriboroumand *et al*., who synthesized AgNps mediated by pomegranate rind. ^4^ The zeta potential was found to be −37 mV to −32 mV and a PDI value of 0.25. It showed a narrow particle size distribution with good stability.

### X-ray diffraction analysis

**Figure 3.**
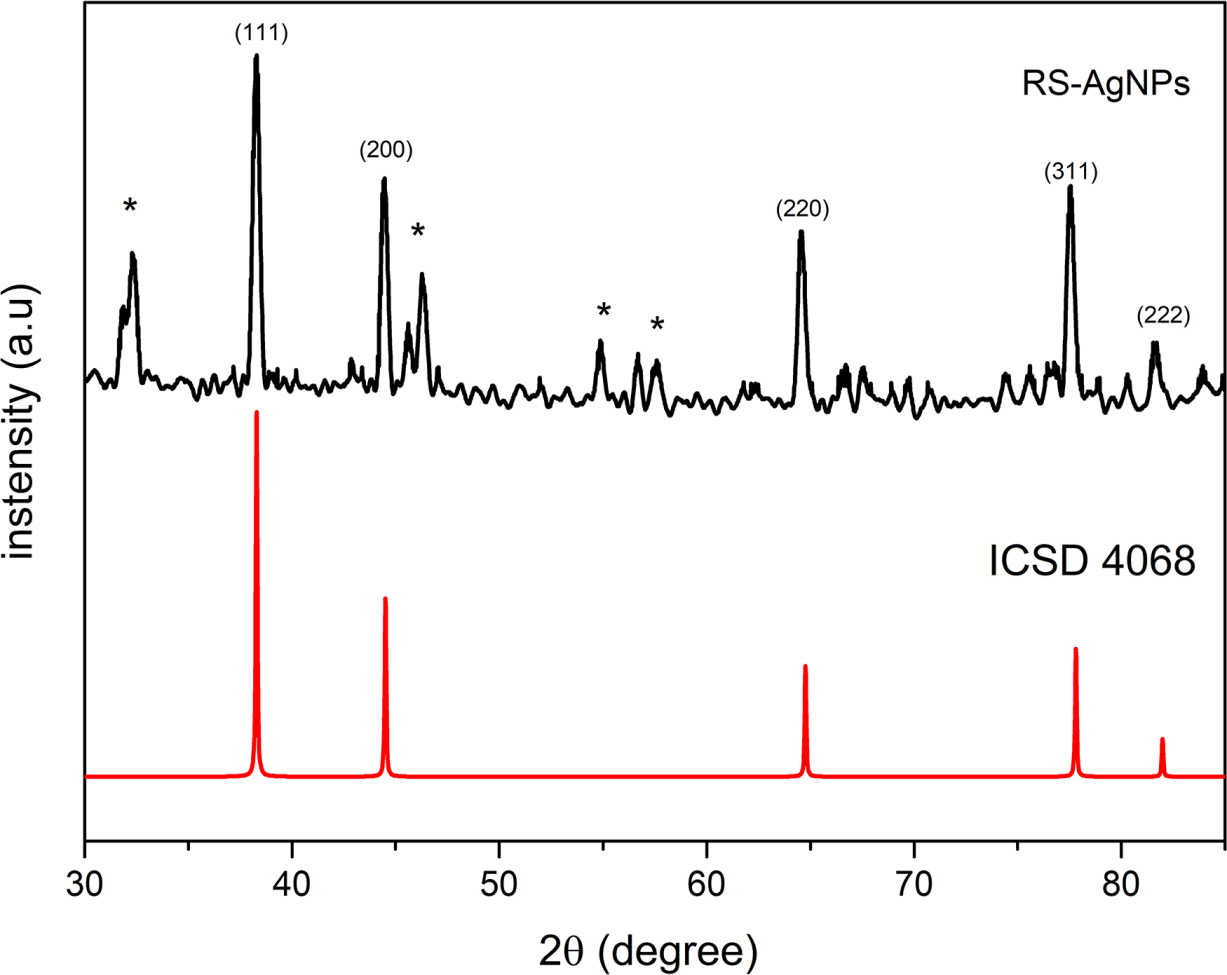
X-ray diffraction of *Rhizophora stylosa* mediated silver nanoparticles. (*) bio-organic crystallization in the plant extract (Khader *et al*., 2019).

X-ray diffraction (XRD) analysis was carried out in order to investigate the crystallinity of as-synthesized RS-AgNps. The diffraction pattern ( Figure 3) showed diffraction peaks that are well resolved at an angle of 2θ at 38.28, 44.45, 64.53 and 77.53, which correspond to (111), (200), (220), (311) of face-centered cubic structure of metallic silver (ICSD No. 4068). These results confirmed UV-Vis spectroscopy and the TEM analysis result, which showed that AgNps have been successfully obtained by the mediation of RS leaf extract. Some other peaks (marked by asterisks*) were observed in the diffraction pattern. It is assumed that these referred to the presence of reducing, and capping agent on the surface of the AgNps and the additional diffraction peaks in the XRD pattern were due to bio-organic crystallization in the plant extract. ^27^ These results were similar to those found in AgNps synthesized through mediation of other marine plants i.e. *Ecklonia cava* algae extract. ^5^ This happened in the use of other marine plants like *Rhizophora stylosa* as well.

### Antibacterial activity

Silver nanoparticles with sizes <100 nm have been of great concern to researchers due to their small particle size and high surface area properties. ^28^ In this study, the antimicrobial activity of RS-AgNps was tested against two types of bacteria i.e. *Escherichia coli* and *Staphylococcus aureus* as representatives of Gram negative and Gram-positive bacteria, which were commonly used to evaluate the activity of nanoparticles in previous studies. ^29, 30^ The antibacterial testing method uses the agar diffusion method. Amoxcillin and distilled water were used as positive and negative controls, respectively. The test was run in duplicate, and all data presented were averaged.

**Figure 4.**
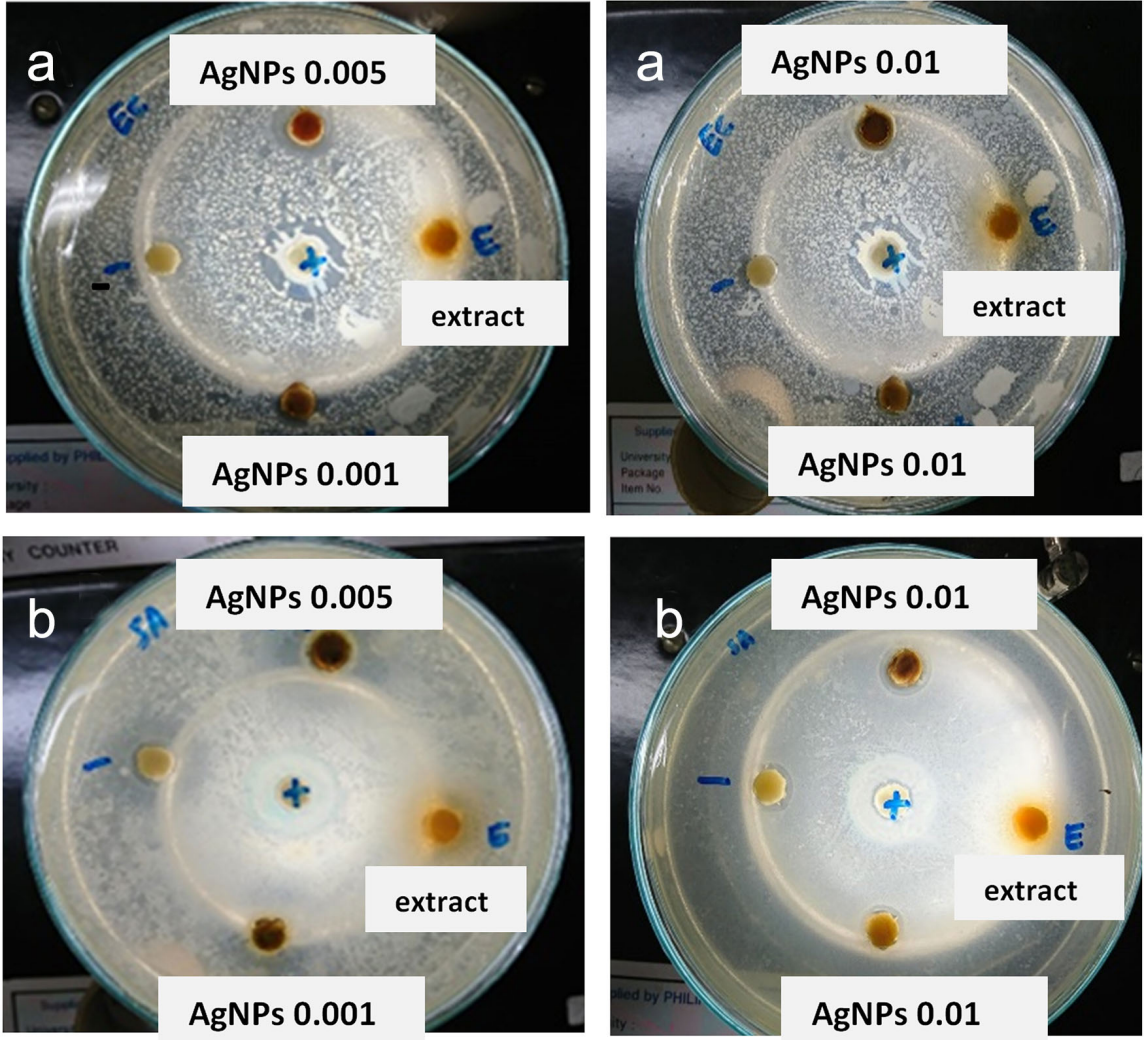
Antibacterial activity of silver nanoparticles against: a) *Escherichia coli* and b) *Staphylococcus aureus.*

The results showed that the three variations in the AgNp concentration significantly affected the inhibition zone. As shown in Table 3 and Figure 4, the area of restriction for a concentration of 0.001 M; 0.005 M and 0.01 M RS-AgNps respectively, are 5.5 mm, 7.2 mm, and 5.1 mm for *E. coli* and 4.3 mm for *S. aureus* bacteria. A higher zone of inhibition was found in the 0.005 M concentration of AgNps against *E. coli* and *S. aureus.*

**Table 3.** Diameter of inhibition zone of synthesized silver nanoparticles (RS-AgNps) against *Escherichia coli* and *Staphylococcus aureus.*

Samples	Inhibition zone (mm) against bacterial strains	
	*E. coli*	*S. aureus*
AgNps 0.001 M, 3% extract	5.5	4.3
AgNps 0.005 M, 3% extract	7.2	4.3
AgNps 0.01 M, 3% extract	5.1	4.3
RS leaf extract	3.8	4.1
Amoxicillin (+)	16	15

Generally, it was observed that as-synthesized AgNps showed a greater inhibitory zone diameter against *E. coli.* than *S. aureus.* These results suggest that RS-AgNps is more specific against Gram negative bacteria than against Gram positive bacteria. The Gram-positive bacteria have a thick layer of peptidoglycan (80 nm) in the cell wall, and this zone has covalent bonds with teichoic and teichuronic acids, whereas Gram negative bacteria have a thin peptidoglycan layer (~8 nm thick) with a lipopolysaccharide external membrane (1–3 μm thick). Another possible cause for their vulnerability to nanoparticles (NP) are these bacteria are coated with lipopolysaccharides, which are negatively charged. These negative loaded molecules are closer to positive ions, which are mostly released through NP leading to ion uptake and increased intracellular damage. NP exhibit their antibacterial activity by different pathways, which are summarized as a combination of ROS production, altered gene regulation, cell wall penetration, and binding metabolites, among other processes. ^31^

In addition, the region of maximum inhibition of AgNps was observed based on the TEM results. As corresponding to the average diameter estimated by TEM analysis, there is a correlation between mean diameter of NP and its antibacterial activity. The resulting smaller-sized particles (22 nm) have a larger inhibitory zone, as well as stable colloidal AgNps. Choi and Hu (2008) reported that this phenomenon was due to the easiness of the small-sized NP to penetrate into the bacteria cell. In addition, smaller NP have a larger surface area, resulting in a larger interaction site of NP and bacteria. ^32^

According to Aromal and Philip (2012), the mechanism of antibacterial activity of metallic NP is highly dependent on the interaction or bond between NP and compounds in the bacteria cell wall. Firstly, the metallic nanoparticles penetrate into the cell and then interfere with cell metabolism, especially with the organelle involved in protein synthesis. ^33^ From this study, it can be concluded that RS-AgNps may be developed as an antimicrobial agent.

Other types of mangroves have also been used as reducing agents for the synthesis of AgNps against pathogenic bacteria including *E. coli and S. aureus*, such as *Ceriops tagal*, ^34^ *Exoecaria agallocha*, ^35^ *Sonneratia apetala*, ^32^ and *Rhizophora mucranata*. ^33^

## Conclusion

Silver nanoparticles with a particle size range of 5–87 nm or an average of 22 nm have been successfully synthesized with a wavelength of 403–443 nm by bioreduction using leaf extracts of the *Rhizophora stylosa* mangrove plant. The results of UV-Vis spectroscopy and TEM analysis showed the formation of smaller sized nanoparticles. DLS, zeta potential and index polydispersion analysts have demonstrated the stability of nanoparticles within a month of the reaction. The influence of reaction time, silver nitrate dissolution concentration and effect of extract amount showed a joint relationship. A silver nitrate concentration of 0.001 M and 0.005 M was able to produce smaller nanoparticles and the optimal concentration of extract used was 3% (v/v). Antibacterial activity showed that silver nanoparticles mediated by RS mangroves have biomedical potential.

## Data availability

All data underlying the results are available as part of the article and no additional source data are required.
