# Supplementary material for: Marine plant mediated green synthesis of silver nanoparticles using mangrove Rhizophora stylosa: Effect of variable process and their antibacterial activity
Source: F1000Res. 2022 May 16;10:768. Originally published 2021 Aug 6. [Version 2] doi: 10.12688/f1000research.54661.2 (PMC10285325; doi:10.12688/f1000research.54661.2)
Supplement: Peer review history

# Peer review: Reviewer response for version 2

**DOI:** 10.5256/f1000research.133904.r172461

**Published:** 2023-06-15

**Authors:** Muberra Andac

**Affiliations:** 1 Department of Chemistry, Department of Nanoscience and Nanotechnology, Ondokuz Mayis Universitesi, Samsun, Samsun, Turkey

The manuscript entitled "Marine plant mediated green synthesis of silver nanoparticles using mangrove *Rhizophora stylosa*: Effect of variable process and their antibacterial activity." was reviewed. In this manuscript, silver nanoparticles (AgNPs) were synthesized using *Rhizophora stylosa* (RS) extract as a reducing and stabilizing agent. The synthesized AgNPs were then applied for their antibacterial activity. The structural, morphological and optical properties of the synthesized AgNPs were characterized using UV-Vis spectrophotometry, X-ray diffraction (XRD), Dynamic Light Scattering (DLS) zetasizer and Transmission Electron Microscopy (TEM).

 The AgNPs were found to be spherical, with average particle sizes of 5-87nm. The antibacterial activity was tested on *Escherichia coli* and *Staphylococcus aureus*. This manuscript can be Approved but I have some major remarks before it can be indexed:

1. The novelty of the work is not clear. The introduction can be improved by providing a more critical discussion of recently published papers.
2. FT-IR Analysis must be included in the results and discussion part. All FT-IR bands are clearly identified and discussed in the text.
3. The author should confirm the bio-components that still attached on AgNPs surface.
4. In figure 2, the author calculates particle size here need a discussion with XRD crystallite size, so author carefully calculate the crystallite size of the prepared nanomaterial.
5. In "Result and Discussions" section, authors should add enough references to support results of their works. All the findings of the current work need to be compared and discussed with the results of other researchers finding.

Is the work clearly and accurately presented and does it cite the current literature?

Partly

If applicable, is the statistical analysis and its interpretation appropriate?

Partly

Are all the source data underlying the results available to ensure full reproducibility?

Partly

Is the study design appropriate and is the work technically sound?

Partly

Are the conclusions drawn adequately supported by the results?

Partly

Are sufficient details of methods and analysis provided to allow replication by others?

No

Reviewer Expertise:

Synthesis and characterization of nanopartcles

I confirm that I have read this submission and believe that I have an appropriate level of expertise to confirm that it is of an acceptable scientific standard, however I have significant reservations, as outlined above.

# Peer review: Reviewer response for version 2

**DOI:** 10.5256/f1000research.133904.r172459

**Published:** 2023-05-22

**Authors:** Buzuayehu Abebe

**Affiliations:** 1 Department of Applied Chemistry, School of Applied Natural Science, Adama Science and Technology University, Adama, Oromia, Ethiopia

This manuscript synthesises silver nanoparticles with a particle size range of 5–87 nm using leaf extracts of the *Rhizophora stylosa* mangrove plant. The formation of smaller AgNps and physical information was characterized by techniques such as DLS, UV-vis, and TEM. The application potential of the synthesized AgNps was tested for antibacterial activity, although it showed less activity. Interesting points are raised with the following amendments before indexing:

- Dynamic Light Scattering in the Abstract: The first letters should be small.
- Once the silver nanoparticles are abbreviated as "AgNps," it should be written throughout the manuscript instead of "silver nanoparticles" again and again.
- The HRTEM analysis should be included to confirm the AgNps from the lattice fringes and d-spacing values.
- The antibacterial activity (zone of inhibition) value is very small compared to the literature review. Would you give a strong interpretation of this?
- The authors should compare the small zone of inhibition presented with the antibacterial activity with the literature and confirm that their work is the best.
- Even though the work is designed appropriately, its soundness will be reduced by less antibacterial activity. Thus, the authors should fill this gap either by doing the experiment again or doing some literature survey on related.

The conclusion is good.

Is the work clearly and accurately presented and does it cite the current literature?

Partly

If applicable, is the statistical analysis and its interpretation appropriate?

Not applicable

Are all the source data underlying the results available to ensure full reproducibility?

Yes

Is the study design appropriate and is the work technically sound?

Partly

Are the conclusions drawn adequately supported by the results?

Yes

Are sufficient details of methods and analysis provided to allow replication by others?

Yes

Reviewer Expertise:

Nanomaterials

I confirm that I have read this submission and believe that I have an appropriate level of expertise to confirm that it is of an acceptable scientific standard, however I have significant reservations, as outlined above.

# Peer review: Reviewer response for version 2

**DOI:** 10.5256/f1000research.133904.r138010

**Published:** 2022-05-18

**Authors:** Manoj Kumar Choudhary

**Affiliations:** 1 Nanomaterial Research Laboratory, Department of Chemistry, Guru Nanak National College, Ludhiana, India

Authors have revised the manuscript very well. It may be Approved in the present form for indexing.

Is the work clearly and accurately presented and does it cite the current literature?

Partly

If applicable, is the statistical analysis and its interpretation appropriate?

Yes

Are all the source data underlying the results available to ensure full reproducibility?

Yes

Is the study design appropriate and is the work technically sound?

Partly

Are the conclusions drawn adequately supported by the results?

Yes

Are sufficient details of methods and analysis provided to allow replication by others?

Yes

Reviewer Expertise:

Synthesis, Characterization and Environmental Applications of metal nanoparticles, carbonaceous materials and photocatalytic materials

I confirm that I have read this submission and believe that I have an appropriate level of expertise to confirm that it is of an acceptable scientific standard.

# Response

**Published:** 2022-05-19

**Authors:** Hilfi Pardi

Thank you to the reviewer (Manoj Kumar Choudhary) who has provided input on our manuscript, hopefully this can improve the quality of our manuscript.

# Peer review: Reviewer response for version 1

**DOI:** 10.5256/f1000research.58166.r122119

**Published:** 2022-02-18

**Authors:** Manoj Kumar Choudhary

**Affiliations:** 1 Nanomaterial Research Laboratory, Department of Chemistry, Guru Nanak National College, Ludhiana, India

1. The theory should be more explanatory and well written to improve the readability of this article.
2. Incorrect inter-relation between the data in the tables and the statements in the text.
3. Lack of consideration of reference materials as the authors of this article directly give statement rather than giving particular reference to that statement.
4. A statement has been given in the article “ *these compounds show extraordinary pharmacological activities and are linked with the presence of –OH*” What does it referred to?
5. In the results and discussion part, the UV-Vis spectrum of synthesized nanoparticles is not clearly explained and hence unjustified.
6. In the discussion of table 1 (in results and discussion part), the relation between the size of Nps, wavelength and concentration of extract should be properly explained. Authors may follow a recent article of mine titled, “Evaluation of the kinetic and catalytic properties of biogenically synthesized silver nanoparticles” ^1^.
7. The statement made by authors “ *Ag Nps with addition of 3% leaf extract tend to have less wavelength shift than those of 1% and 5%*” needs more elaborated explanation with proper justification and latest reference to support it.

Is the work clearly and accurately presented and does it cite the current literature?

Partly

If applicable, is the statistical analysis and its interpretation appropriate?

Yes

Are all the source data underlying the results available to ensure full reproducibility?

Yes

Is the study design appropriate and is the work technically sound?

Partly

Are the conclusions drawn adequately supported by the results?

Yes

Are sufficient details of methods and analysis provided to allow replication by others?

Yes

Reviewer Expertise:

Synthesis, Characterization and Environmental Applications of metal nanoparticles, carbonaceous materials and photocatalytic materials

I confirm that I have read this submission and believe that I have an appropriate level of expertise to confirm that it is of an acceptable scientific standard, however I have significant reservations, as outlined above.

## References

[1] : Evaluation of the kinetic and catalytic properties of biogenically synthesized silver nanoparticles. *Journal of Cleaner Production* .2018;198: 10.1016/j.jclepro.2018.09.015 882-890 10.1016/j.jclepro.2018.09.015
